# Lights and Shadows in the Use of Mesenchymal Stem Cells in Lung Inflammation, a Poorly Investigated Topic in Cystic Fibrosis

**DOI:** 10.3390/cells9010020

**Published:** 2019-12-19

**Authors:** Anna Caretti, Valeria Peli, Michela Colombo, Aida Zulueta

**Affiliations:** 1 Department of Health Sciences, University of Milan, 20142 Milano, Italy; anna.caretti@unimi.it (A.C.); valeria.peli@unimi.it (V.P.); michela.colombo@ndcls.ox.ac.uk (M.C.); 2 Haematopoietic Stem Cell Biology Laboratory, Medical Research Council (MRC) Weatherall Institute of Molecular Medicine (WIMM), University of Oxford, Oxford OX39DS, UK

**Keywords:** inflammation, mesenchymal stem cells, cystic fibrosis, extracellular vesicles, anti-inflammatory therapy

## Abstract

Mesenchymal stem cells (MSCs) are multipotent non-hematopoietic stem cells residing in many tissues, including the lung. MSCs have long been regarded as a promising tool for cell-based therapy because of their ability to replace damaged tissue by differentiating into the resident cell and repopulating the injured area. Their ability to release soluble factors and extracellular vesicles has emerged as crucial in the resolution of inflammation and injury. There is a growing literature on the use of MSCs and MSC secretome to hamper inflammation in different lung pathologies, including: asthma, pneumonia, acute lung injury (ALI), pulmonary hypertension, and chronic obstructive pulmonary disease (COPD). However, their potential therapeutic role in the context of Cystic Fibrosis (CF) lung inflammation is still not fully characterized. CF morbidity and mortality are mainly due to progressive lung dysfunction. Lung inflammation is a chronic and unresolved condition that triggers progressive tissue damage. Thus, it becomes even more important to develop innovative immunomodulatory therapies aside from classic anti-inflammatory agents. Here, we address the main features of CF and the implications in lung inflammation. We then review how MSCs and MSC secretome participate in attenuating inflammation in pulmonary pathologies, emphasizing the significant potential of MSCs as new therapeutic approach in CF.

## 1. Cystic Fibrosis Lung Inflammation and Current Therapies

Cystic fibrosis (CF) is the most common lethal genetic disease in Caucasian populations. It is a multisystemic disease characterized by a dysregulated ions flux through the epithelia, and it is caused by mutations in the gene encoding for cystic fibrosis transmembrane conductance regulator (CFTR) channel. The multisystemic feature of CF is due to the ubiquitous localization of CFTR, which is expressed on the apical membrane of epithelial secretory cells. For this reason, CF involves several organs such as lungs, kidneys, gut, liver, and pancreas. The condition of CF adult patients is further complicated by several age-related comorbidities such as diabetes, nephropathy, coronary artery disease, and increased risk of gastrointestinal cancer [1]. However, despite this growing variety of comorbidities affecting CF patients, morbidity and mortality are mainly due to lung disease. Indeed, respiratory failure leads to premature death in about 90% of people affected by CF [2].

CF pulmonary manifestations are characterized by progressive damage of pulmonary architecture, because of persistent infection and chronic inflammation. Deficiency of CFTR functions leads to altered ion homeostasis and water flux on pulmonary epithelial surface, resulting in accumulation of dehydrated, thick and viscous mucus, which tightly adheres to epithelial airways surface and strongly impairs mucociliary clearance [3,4]. This in turn promotes airways obstruction and hinders elimination of pathogens. Moreover, pH of CF airways fluid is eight-fold more acidic than non-CF [5], further contributing to inappropriate microbial clearance because of inactivation of mucus antimicrobial peptides [6]. Finally, recurrent and unresolved microbial colonization promotes a chronic state of infection. Indeed, *Staphylococcus aureus* and *Haemophilus influenza* colonize CF lungs very early in patients’ life [7], while during adolescence and adulthood, *Pseudomonas aeruginosa* and other bacterial/non-bacterial microorganisms, worsen pulmonary conditions [7]. Pathogens colonization induces an intense inflammatory response, which is ineffective in eradicating infections and perpetuates over time. The unresolved chronic inflammation becomes harmful and finally compromises respiratory function. Though CF inflammatory condition is characterized by a massive neutrophil influx, driven by chemotactic signals, these neutrophils fail in resolving infection, accumulate in the pulmonary tissue [8,9] and secrete pro-oxidants mediators and proteases that exacerbate the inflammatory milieu. High levels of neutrophil elastase (NE) in CF sputum correlate with lung disease progression [10]. NE affects the airways remodeling, impairs mucociliary clearance, and activates matrix metalloproteinases [11,12], thus contributing to destroy the architecture of airways epithelium [13]. CF patients display altered mechanisms of phagocytosis to eliminate dead neutrophils, which further fuel chronic inflammation by accumulating and releasing great amounts of intracellular material. CF airways are characterized by an imbalance between pro- and anti-inflammatory mediators [14,15,16]. Bronchoalveolar lavage (BAL) of infants younger than 3 years old, revealed high concentrations of IL-8 even in the absence of any detectable infection [17,18], suggesting that inflammation is an intrinsic feature of CF airways, independent from microbial colonization. Moreover, it has been demonstrated that CF patients have an impaired capacity to biosynthesize specialized pro-resolving lipid mediators (SPMs), like lipoxins, maresins, protectins, and resolvins, because of the altered metabolism of arachidonic acid and docosahexaenoic acid [16,19,20]. This inefficient production of bioactive lipids significantly contributes to the defective resolution of inflammation in CF since SPMs restore functions that are altered in CF airway such as stimulation of macrophage bactericidal activity [21,22], stimulation of epithelial chloride secretion and airway surface liquid layer height increase [23,24], and enhancement of epithelial cohesion and repair [25,26].

Because of the significant impact of the lung dysfunction affecting CF patient, a great deal of effort has been put in identifying various therapeutic strategies toward lung manifestations. Almost 2000 mutations in the CFTR gene have been identified allowing the development of novel therapies targeting specific molecular basic defects (for a comprehensive review please refer to Pranke et al. 2019 [27]). Some of these mutation specific strategies aim to restore mRNA levels, correct CFTR folding and trafficking to the apical plasma membrane (correctors) or increase the CFTR channel function (potentiators). However, molecules targeting basic defects of CFTR protein are specific and address only patients carrying a specific mutation/class of mutation. Other therapies that are in preclinical development are not mutation specific and include gene therapy to edit the genome and stem cell therapy to repair the airway tissue. Indeed, CF being a monogenic disease is an ideal candidate for gene therapy. Genetic therapies using viral and non-viral vectors, the use of CRISPR/Cas9 approach, antisense oligonucleotides and RNA- mediated therapy are promising tools for CF treatment that are been tested in preclinical studies [27]. With the discovery of the induced pluripotent stem cells (iPSCs), stem cells therapy in combination with CRISPR/Cas9 technology theoretically allow to re-graft the lung niches and repopulate the respiratory cells. However, important limitations have hindered CFTR gene therapy such as the expensiveness, the difficult delivery to the lungs and the necessity of repeated administration. Moreover, time and labor efforts together with the complication to obtain fully differentiated lung-specific cell subsets limit the efficacy of stem cell-based therapy. Lung transplantation is the only therapeutic life-saving option for patients with end-stage lung disease. Although it has evolved over the last decades, the median survival after surgery for CF patients is 9.5 years as reported by the International Society for Heart and Lung Transplantation [28]. Lung transplantation is a high-risk procedure and it implies transplant-associated complications and graft dysfunctions [29,30]. Therefore, the benefit/risk rate of this therapeutic approach is still controversial.

While no cures for CF are known, currently available therapies target symptoms. Given the key role of chronic inflammation in worsening CF lung pathological condition, it is important to consider pulmonary inflammation as a therapeutic target. Indeed, counteracting inflammation could slow down pulmonary decline and preserve respiratory capacity, improving the quality of patients’ life. Several anti-inflammatory strategies have been investigated in the last 20 years though few of them have been translated into clinical application (see Table 1). The use of anti-inflammatory treatment, by aerosol or orally, is limited by the undesired side effects such as intolerance to glucose, diabetes, growth impairment, cataracts, gastrointestinal toxicity [31,32]. Therefore, the need to develop innovative immunomodulatory therapies aside from the classic anti-inflammatory agents is compelling. Though in its early beginning, accumulating evidences support the hypothesis that mesenchymal stem cells (MSCs) treatment represents a novel strategy to be investigated in CF.

## 2. Mesenchymal Stem Cells: Principal Characteristics and Regenerative Properties

MSCs were originally discovered in bone marrow by Friedenstein and colleagues in 1968 [75]. Successively, MSCs have been isolated from a variety of tissues, such as umbilical cord blood, Wharton’s jelly, placental, and adipose tissues [76,77,78]. Likewise, MSCs have been identified in the lung tissue as important components of the parenchymal progenitor cell niche, orchestrating organ homeostasis, and injury repair [79]. MSCs represent a subpopulation of stem cells in the context of mesenchymal stromal cells, according to the current criteria of isolation that produce heterogeneous, non-clonal cultures of stromal cells containing stem cells with different multi potential properties, committed progenitors, and differentiated cells. Stromal cultures obtained from various tissues that contain a subpopulation of stem cells are used for therapeutic purposes, since they might be effective in the treatment of several diseases [80]. In 2006, the Mesenchymal and Tissue Stem Cell Committee of the International Society for Cellular Therapy, recommended minimal criteria for their characterization [81]. Because of the low expression of MHC class I and no expression of MHC class II, MSCs are considered immunopriviledged cells, able to avoid immunosurveillance [82].

Homing and migration are other peculiar characteristics of MSCs. Many studies have demonstrated that when systemically administered, MSCs are recruited to the injured sites to exert their therapeutic effects. Once engrafted, they replace damaged tissue through differentiation into the resident cells and repopulation of injured area [83,84,85]. For these reasons, they have been regarded as an encouraging tool for regenerative medicine. Concerning pulmonary tissues, increasing evidences support MSCs regenerative properties. Indeed, they were able to engraft and to differentiate into lung epithelial cells under experimental conditions such as irradiation, bleomycin, or LPS-induced injury [86,87,88]. A systematic review of seventeen studies on rodents models of bleomycin-induced pulmonary fibrosis, describes the beneficial effects of MSCs, mostly derived from bone marrow, in improving alveolar injury and in decreasing lung fibrosis and collagen content [86]. Aged Balb/C mice infected with highly pathogenic H5N1 and H7N9 influenza viruses, show impaired alveolar fluid clearance and protein permeability. The pathological phenotype was reduced by injected intravenous administration of human MSCs derived from bone marrow [89]. In a mouse model of acute respiratory distress syndrome (ARDS) induced by LPS, Gupta N. and colleagues demonstrated that systemic administration of MSCs significantly improved alveolar injury [90]. MSCs-treated mice showed attenuated fibrosis and reduced collagen accumulation consequent to irradiation-induced lung injury [91].

However, a growing body of evidence suggests that MSCs ability to repair and improve injured tissues function do not necessarily require significant engraftment or differentiation rate. The low survival rate of the transplanted MSCs in the recipient tissue may be affected by culture conditions or by cell death caused by lack of proper interconnections, thus representing a great limitation to their use [92]. In fact, numerous preclinical studies have shown that the engraftment rate is very low, near 5% [85,93]. This supports the hypothesis that the regenerative ability may be mediated by their secretome. Indeed, MSCs paracrine effects are extensively studied, and it is widely accepted that their secretome is majorly responsible for the intercellular crosstalk between MSCs and targeted cells [94,95,96]. Bioactive factors secreted by MSCs are known to mediate some of the functions of MSCs, such as the immunomodulatory, anti-inflammatory, anti-oxidant, and anti-apoptotic activity. They also contribute to tissue regeneration, angiogenesis, and clearance of microorganisms. Other than the soluble factors, MSCs also secrete different types of extracellular vesicles (EVs) accounting for their secretome and supporting all their therapeutic effects [97,98,99,100,101].

Despite the abovementioned regenerative properties of MSCs, this review focuses on the immunomodulatory and anti-inflammatory effects of MSCs on lung inflammatory pathologies, with a peculiar attention toward CF, that is still a poorly investigated field.

## 3. Mesenchymal Stem Cells in Lung Inflammation

MSCs can modulate the inflammatory response during pulmonary inflammation restoring the balance between the cytokine network and other humoral or cellular effectors of the immune system. Several preclinical studies demonstrated that the administration of MSCs stimulates the reduction of pro-inflammatory cytokines and the increase of anti-inflammatory markers and cytokines in lung tissues [102,103,104,105]. Moreover, MSCs treatment promoted neutrophil-mediated phagocytosis in vitro and in vivo and thus bacterial clearance [106] as well as specialized pro-resolving lipid mediators release, particularly D-series resolvins [107] in a model of sepsis. In *Escherichia coli*-induced pneumonia, the intratracheal MSCs treatment was as effective as intravenous therapy decreasing the infiltration of neutrophils, reducing lung bacterial load and suppressing inflammation [108]. Pedrazza and colleagues found that MSCs reduced inflammation, oxidative damage, and consequently decreased the release of neutrophil extracellular traps in a LPS-induced acute lung injury (ALI) model [109]. They also demonstrated that treatment with MSCs was able to reduce COX-2 and NF-κB and to inhibit the MAPK pathway activation, modulating the inflammatory response during sepsis [110]. MSCs can promote macrophage differentiation to M2 phenotype contributing to the resolution of inflammation, and conversely, to M1 phenotype leading to augmented phagocytic activity [102,111]. Recent studies have demonstrated that MSCs can also transfer functional mitochondria to the damaged cells reducing oxidative stress in lung injury [112,113]. Keeping in view that oxidative stress-induced mitochondrial dysfunction can contribute to inflammation, transfer of mitochondria could be consider another anti-inflammatory effect of MSCs. Indeed, mitochondria delivered from iPSC-MSCs to airway smooth muscle cells alleviate airway inflammation and hyper responsiveness in COPD models of human lung cells and mouse lungs [112]. Moreover, mitochondrial transfer from iPSC-MSCs to epithelial cells through tunneling nanotubes attenuated the mitochondrial dysfunction and ameliorated asthma inflammation in mice [113].

The increasing number of MSCs-based clinical trials in lung pathologies, either complete or ongoing, supports the feasibility of this therapeutic approach. Indeed, MSCs are easy to isolate and expand in culture, they have inherent low immunogenicity and limited risk of tumorigenicity and are easy to be administered. According to the US National Institutes of Health (http://www.clinicaltrials.gov/) until December 2019, almost 10% of MSCs-based clinical trials in lung diseases were performed using autologous MSCs. In 50% of allogeneic MSCs-based clinical trials, bone marrow MSCs from healthy donors were administered to patients, whereas almost 40% of the remaining trials were carried out with perinatal MSCs. Among perinatal sources cord blood was the most tested, but also umbilical cord- and placenta-derived MSCs were studied. MSCs were administered by intravenous infusion (as shown by half of clinical trials), single intratracheal administration or via bronchoscopy among others. The dosage of administration largely varied from 1–5 × 10^6^ to 10–20 × 10^6^ cells/kg or 100–200 × 10^6^ cells/infusion.

However, MSCs-based therapy faces significant hurdles in its translation to clinic as elegantly described by Galipeau J. [114]. The long-term benefits of MSCs are not currently clear and the final outcome is highly dependent on patient inter-variability [115]. Data arising out of MSCs-based clinical trials demonstrate the variability of the use of this therapy in terms of MSCs source, route, timing, and dosage of administration. As very recently reviewed by Zanoni M. et al. [87], the lack of consensus on these parameters (route, timing and dosage) together with the paucity of standardized methods for MSCs harvesting, request further studies in order to include MSCs therapy in the clinical practice in the near future. Moreover, donor variance, senescence, cryopreservation, and sources are among the main variables that can affect cellular therapies based on MSCs [116,117]. Specifically, the MSCs source is an important point to be addressed since it may result in different effects in respiratory diseases. In a rat model of ARDS, Silva J.D. and colleagues observed that both bone marrow- and adipose tissue-derived MSCs yielded greater beneficial effects in term of reduced expression of pro-inflammatory cytokines than lung- derived MSCs [118]. On the other hand, the potential risk of unpredictable cell growth and differentiation of MSCs-based therapy is still a major concern [119].

The low rates of engraftment suggest that the therapeutic benefits of MSCs mostly derive from the paracrine effects of their secretome. Thus, cell-free approach using the MSCs secretome arises as a powerful tool against lung inflammation with the same positive effects of MSCs, but with less unwanted secondary effects as recently reviewed [120,121,122].

## 4. Mesenchymal Stem Cells-Derived Conditioned Medium in Lung Inflammation

The potential of MSCs-conditioned media (CM) to mimic the anti-inflammatory mechanisms of the parental MSC has been proved in several studies using different animal models of lung injury. In acute and chronic asthma, intranasal instillation of bone marrow MSCs-CM reduced lung levels of the inflammatory cytokines IL-4 and IL-13 and increased levels of IL-10. MSCs-CM prevented airway hyper responsiveness, smooth muscle thickening, and peribronchial inflammation via adiponectin, an anti-inflammatory adipokine found in the CM [123]. Pretreatment with MSCs-CM protected against lung ischemia-reperfusion (IR) injury in a rat model with a significant reduction in proinflammatory cytokines, a decrease in infiltrating inflammatory cells, and an increase in M2-like macrophages and regulatory T cells [124]. Shen and colleagues studied the protective effects of bone marrow MSCs-CM on bleomycin induced lung injury and fibrosis, both in vitro (A549 alveolar epithelial cells) and in vivo (rat model). Following MSCs-CM treatment, A549 cells were significantly protected from bleomycin-induced apoptosis while bleomycin-challenged rat lungs exhibited a reduction in inflammation, fibrotic scores, collagen deposition, and cell apoptosis [125]. In a rat model of COPD, the administration of MSCs-CM attenuated the cigarette smoke-induced emphysema and increased the number of small pulmonary vessels as well as the MSCs [126].

A main limitation in the use of MSCs-CM is the relatively rapid degradation of bioactive molecules in extracellular medium [119]. Moreover, the preconditioning process, the optimal dose, timing and route of administration lack fully standardized protocols. Lately, EVs have attracted the scientific community’s attention for their role in transporting both bioactive molecules, thus preventing them from hydrolysis, and DNA, thus avoiding mutations [127]. Given their potential, EVs arise as a good candidate for cell-free therapy in regenerative medicine.

## 5. Mesenchymal Stem Cells-Derived EVs in Lung Inflammation

EVs are diverse populations of small, lipid-enclosed, cell-derived particles released by almost all cellular types to promote cell-cell communication [128,129,130]. They function as shuttle vehicles toward target cells for their content, but also for soluble factors in the microenvironment that can bind to their membranes [131,132]. MSCs release diverse classes of EVs, including exosomes, microvesicles and apoptotic bodies. Several parameters have been used to classify EVs such as the cellular origin, secretory mechanism, size, and surface markers. Nevertheless, most of these parameters are not exclusive to any specific type of EVs [133]. Recent evidences suggest that EVs secreted from MSCs may release bio-active protein, lipid, and nucleic acid cargo to promote functional changes in the recipient injured cells [134,135]. MSCs-derived EVs have been shown to suppress pro-inflammatory processes and reduce pulmonary fibrosis in a variety of experimental models of inflammatory lung diseases, including asthma, pulmonary artery hypertension, ALI, ARDS, and pneumonia by transferring their components.

In a murine model of hypoxia-induced pulmonary hypertension, the intravenous administration of MSCs-derived exosomes inhibited STAT3 signalling and restored the levels of both the miR-17 and the miR-204 superfamily, resulting in the inhibition of vascular remodelling and hypoxic pulmonary hypertension [98]. Zhu and colleagues demonstrated that human bone marrow MSCs-derived EVs were effective in reducing lung inflammation in *Escherichia coli* endotoxin-induced ALI in C57BL/6 mice. The intratracheal delivery of EVs, attenuated the pulmonary edema, the lung protein permeability, the influx of neutrophils, and the expression of macrophage inflammatory protein-2 in the BAL fluid [99]. Results from the same group demonstrated that EVs derived from human MSCs limited the influx of inflammatory cells, cytokines, and bacteria in a murine model of *Escherichia coli* pneumonia. Besides, they found that EVs increased monocyte phagocytosis of bacteria while decreasing inflammatory cytokine secretion in injured alveolar epithelial type 2 cells [136]. Interestingly, EVs isolated from human MSCs were more efficient than those from mouse MSCs in a preclinical model of asthma. The systemic administration of EVs from both sources of MSCs ameliorated the *Aspergillus* hyphal extract-induced increases in airway hyper reactivity and lung inflammation in immunocompetent C57Bl/6 mice [137]. In a model of lung fibrosis, the intravenous treatment with isolated human MSCs-derived exosomes, significantly reduced the extent of monocyte infiltration and expression of inflammatory and pro-fibrotic genes in the lung [127]. Alveolar macrophages pre-treated with MSCs-derived EVs reduced the inflammation and lung injury in LPS-injured mice. This effect was induced by EVs-mediated mitochondrial transfer as reported by Morrison et al. [138]. The anti-inflammatory and immunomodulatory activity of MSCs-derived EVs was found to attenuate lung IR injury in mice. A significant downregulation of proinflammatory cytokines (IL-17, TNF-α, CXCL1, and HMGB1) together with an increase of keratinocyte growth factor, prostaglandin E2, and IL-10 occurred in the BAL fluid from EVs-treated mice [139]. Human MSCs-derived EVs have also been tested in hyperoxic-induced lung injury models using newborn mice [140] and newborn Sprague-Dawley rats [141]. Willis and colleagues found a macrophage phenotype modulation toward an anti-inflammatory M2-like state after the MSCs-exosome treatment, resulting in the amelioration of lung function, pulmonary fibrosis, vascular remodelling, and pulmonary hypertension [140]. Accordingly, in the neonatal rat model, MSCs-derived EVs were as effective as parental MSCs to confer a VEGF mediated-protection against activated macrophages, proinflammatory cytokines, and augmented cell death [141]. Recently, it was demonstrated that MSCs-EVs have anti-influenza and anti-inflammatory properties in a pig model of influenza virus because of RNAs transfer to epithelial cells. EVs inhibited the hemagglutination activity of avian, swine, and human influenza viruses and inhibited influenza virus replication and virus-induced apoptosis in lung epithelial cells [142]. Several evidences indicated that MSCs-derived EVs can carry miRNAs, which have anti-apoptotic properties and can contribute to contrast pulmonary inflammation [143,144,145]. Indeed, in a murine model of sepsis, MSCs promote macrophage polarization to the M2 phenotype not only in vitro but also in lung and liver tissues. These immunomodulatory properties were mediated by the release of the exosomal miR-146a [143]. Apoptosis of epithelial and endothelial cells increases blood vessel wall permeability, which allows neutrophil infiltration and aggravates the pulmonary edema and inflammation. Hence, the delivery of anti-apoptotic miRNAs by MSCs could be a promising approach to ameliorate inflammation. Finally, anti-apoptotic miRNAs miR-30b-3p and miR-21-5p carried by MSCs-derived exosomes conferred protection against ALI and decreased the apoptosis of alveolar epithelial and endothelial cells, respectively [144,145].

One important therapeutic advantage of EVs, is the encapsulation and protection of their cargo from unfavorable conditions, such as changes in pH or degradation in vivo. In fact, MSCs-EVs can be used to deliver to target organs specific compounds which are overexpressed by the MSCs themselves or which can be loaded directly into the EVs. Moreover, they easily enter the systemic circulation and maintain long lasting high concentrations, while the concentration of transfused MSCs drops rapidly [146]. EVs are not self-replicating, thus reducing the risk of tumor formation and can be stored for several months at −20 °C or −80 °C, without cryopreservatives, remaining biologically active.

**Figure 1:**
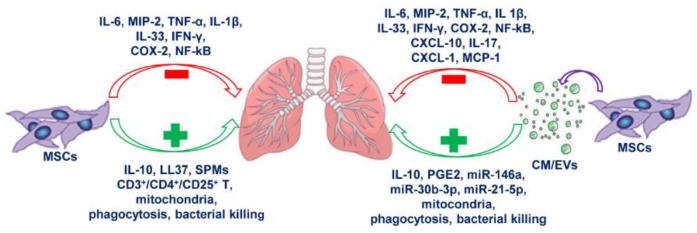
Anti-inflammatory effects of mesenchymal stem cells (MSCs) and secretome on lung inflammation. The scheme represents the main inflammatory mediators modulated by MSCs and secretome in lung diseases. The green color used for the boxes and the arrows indicate anti-inflammatory mediators and functions induced by MSCs and/or MSCs-derived conditioned medium (CM) or extracellular vesicles (EVs). The red color used for the boxes and the arrows indicate pro-inflammatory mediators and functions counteracted by MSCs and/or MSCs-derived CM or EVs. CXCL-1/CXCL-10: chemokine (C-X-C motif) ligand-1/ligand-10; COX-2: cyclooxygenase 2; IFN-γ: gamma interferon; LL37: antimicrobial peptide; MCP-1: monocyte chemoattractant protein 1; MIP-2: macrophage inflammatory protein 2; NF-κB: nuclear factor κB; PGE2: prostaglandin 2; SPMs: specialized pro-resolving lipid mediators; TNF-α: tumor necrosis factor alpha.

Nevertheless, also the use of EVs in clinics has to overcome several limitations such as quantification, scaling up EVs production, delivery route, and effective doses [147]. The heterogeneity of EVs population is another issue that should be carefully addressed together with the great variability of the bioactive compounds content which can be highly modulated by cell culture conditions and stimuli that trigger their release. Despite the extensive use of MSCs and their secretome in different scenarios of lung inflammation, as summarized in Figure 1, their potential has been poorly studied in the context of CF lung inflammation.

## 6. Mesenchymal Stem Cells as Anti-Inflammatory Therapy in Cystic Fibrosis

Bonfield’s group was the first to approach the study of MSCs as anti-inflammatory therapy in CF. In 2013, they reported for the first time that in an in vivo model mimicking CF lung infection and inflammation, human MSCs treatment attenuated pro-inflammatory cytokines levels, weight loss, clinical score, and lung pathology associated with chronic infection with *Pseudomonas aeruginosa*. Their results suggested that the mechanisms supporting the anti-inflammatory and anti-microbial effects involved soluble factors of the human MSCs media, like the cathelicidin LL-37 [71]. In another study using *Pseudomonas aeruginosa* and *Staphylococcus aureus*, they strengthened that the antimicrobial efficacy of human MSCs is due to the release of the antimicrobial peptide LL-37 which slows the rate of bacterial growth [72]. They also demonstrated that the source of the human MSCs (bone marrow or adipose tissue), growth conditions, concentration and time course could impact the overall antimicrobial efficiency. Of interest, in in vitro antimicrobial assays, MSCs with dysfunctional CFTR channel were not as efficient in handling infection as MSCs with fully active CFTR channel, suggesting the same scenario for CF patient’s MSCs as compared to healthy individual’s MSCs [72]. Lately, they investigated the anti-inflammatory properties of human MSCs in both Gram negative (*Pseudomonas aeruginosa*) and Gram positive (*Staphylococcus aureus*) CF murine models. They found that MSCs have the ability of selectively recruiting more lymphocytes than monocytes or neutrophils and of modulating the immune response balance enhancing the expression of anti-inflammatory chemokines IL-6 and CCL2, while decreasing IL-8 expression. They confirmed that inadequate CFTR activity alters the ability of the MSCs to respond to LPS and Gram negative bacteria and it is associated with a deficiency of the anti-inflammatory transcriptional regulator PPARγ, further contributing to inflammation. On the other hand, healthy MSCs were able to reestablish PPARγ expression in CFTR-deficient macrophages as a potential mechanism implicated in the MSCs anti-inflammatory effectiveness in CF [73]. In support of these findings, very recent data from our group demonstrated that human lung MSCs and MSCs-derived EVs could be immunologically altered in CF [148]. In a model of CF-MSCs, CFTR dysfunction associates with an enhanced sphingolipid metabolism leading to the release of EVs that export the excess of pro-inflammatory ceramides to the recipient cells, thus contributing to maintain the unresolved inflammatory status of CF [148].

We also explored the anti-inflammatory effects of EVs, released from human lung MSCs, in CF bronchial epithelial cell models. We found that MSCs-derived EVs down-regulated the transcription and expression of pro-inflammatory cytokines such as IL-1β, IL-8, IL-6 under TNFα-stimulated conditions and partially impaired the nuclear translocation of NF-κB. EVs treatment enhanced the antioxidant defensive response of the CF cells by means of up-regulating heme oxygenase-1 (HO-1) transcription. Noteworthy, we found that MSCs-derived EVs treatment partially restored PPARγ signaling in CF deficient cells, accordingly to the results of Bonfield’s group. Hence, we proposed the modulation of PPARγ axis and its down-stream effectors (NF-κB and HO-1) as a possible mechanism supporting the anti-inflammatory effects of MSCs-derived EVs in CF [74]. Data by our and Bonfield’s group suggest that the lung MSCs compartment could be immunologically impaired in CF, thus contributing to maintain the unresolved pulmonary inflammation. This prompt us to hypothesize that allogeneic MSCs or MSCs-derived EVs administration could represent a novel anti-inflammatory strategy to be investigated. Despite the relatively low abundance of preclinical research concerning the use of MSCs in CF, currently there are two ongoing phase I clinical trials (NCT02866721 and NCT03058068). These are aimed to evaluate the safety, tolerability, and efficacy of the administration of allogeneic MSCs in CF patients and will provide the rationale for new MSCs-related therapeutic approaches.

## 7. Conclusions

While great progresses have been achieved in the study of MSCs, many challenges remain open before their use can be translated in CF clinical therapy. The very high patient inter-variability together with the not completely clear long-term benefits of MSCs may represent a limitation to their use. The preclinical studies reviewed here emphasize the significant potential of MSCs for treating pulmonary inflammatory pathologies, including CF. Continuous efforts should be dedicated to optimize MSCs preparation and administration, dosage, route of administration and to enhance the survival rate of the transplanted MSCs in the recipient tissue.

As it concerns the use of EVs, it becomes necessary to deeply characterize EVs’ population and their bioactive modulators profile, and to better elucidate possible mechanisms underlying their activity. Moreover, the consolidated anti-inflammatory potential exhibited toward multiple inflammatory pulmonary conditions and the very recent results in CF models, encourage to go further with the research in this innovative field.

## Figures and Tables

**Table 1:** Overview of the main cystic fibrosis anti-inflammatory therapies in preclinical or clinical phase.

Compound	Status	References
Systemic corticosteroids	Clinical trials	[33,34,35,36]
Inhaled corticosteroids	Clinical trials	[37]
Ibuprofen	In therapy	[31]
Acebilustat	Clinical trials	NCT02443688
Azithromycin	In therapy	[38,39,40,41]
α1-antitrypsin	Clinical trials	[42,43]
Glutathione	Clinical trials	[44,45,46]
*N*-acetylcysteine	Clinical trials	[47,48,49,50]
Thiocyanate	Preclinical studies	[51]
SB-656933	Clinical trials	[52]
Anakinra	Preclinical studies	[53,54,55]
IL-10	Preclinical studies	[56,57]
Dornase alfa	In therapy	[36]
DHA	Clinical trials	[58,59,60]
LAU-7b	Clinical trials	NCT03265288
Sildenafil	Clinical trials	[61]
Vardenafil	Preclinical studies	[62]
Myriocin	Preclinical studies	[63,64]
Amitriptyline	Clinical trials	[65,66,67]
Miglustat	Preclinical studies	[68]
Ajulemic acid	Clinical trials	[69], NCT02465450
KB001-A	Clinical trials	[70]
Mesenchymal stem cells	Preclinical studies	[71,72,73]
Mesenchymal stem cells-derived EVs	Preclinical studies	[74]
